# Differences in Heart Rate Variability Associated with Long-Term Exposure to NO_2_

**DOI:** 10.1289/ehp.11377

**Published:** 2008-06-20

**Authors:** Denise Felber Dietrich, Armin Gemperli, Jean-Michel Gaspoz, Christian Schindler, L.-J. Sally Liu, Diane R. Gold, Joel Schwartz, Thierry Rochat, Jean-Claude Barthélémy, Marco Pons, Frédéric Roche, Nicole M. Probst Hensch, Pierre-Olivier Bridevaux, Margaret W. Gerbase, Urs Neu, Ursula Ackermann-Liebrich, the SAPALDIA Team

**Affiliations:** 1 Institute of Social and Preventive Medicine, University of Basel, Basel, Switzerland; 2 Department of Community Medicine and Primary Care, University Hospitals, Geneva, Switzerland; 3 Department of Environmental and Occupational Health Sciences, University of Washington, Seattle, Washington, USA; 4 Harvard Medical School, Brigham and Women’s Hospital, Boston, Massachusetts, USA; 5 Department of Environmental Health, Harvard School of Public Health, Boston, Massachusetts, USA; 6 Division of Pulmonary Medicine, University Hospitals, Geneva, Switzerland; 7 Laboratoire de Physiologie Clinique et de l’Exercice, Université Jean Monnet, Saint-Etienne, France; 8 Service of Pulmonology, Ospedale Regionale, Lugano, Switzerland; 9 Molecular Epidemiology/Cancer Registry, University of Zürich, Zürich, Switzerland; 10 ProClim, Swiss Academy of Sciences, Bern, Switzerland

**Keywords:** air pollution, autonomic nervous system, cardiovascular diseases, cohort study, heart rate variability, long-term exposure, nitrogen dioxide, sex

## Abstract

**Background:**

Heart rate variability (HRV), a measure of cardiac autonomic tone, has been associated with cardiovascular morbidity and mortality. Short-term studies have shown that subjects exposed to higher traffic-associated air pollutant levels have lower HRV.

**Objective:**

Our objective was to investigate the effect of long-term exposure to nitrogen dioxide on HRV in the Swiss cohort Study on Air Pollution and Lung Diseases in Adults (SAPALDIA).

**Methods:**

We recorded 24-hr electrocardiograms in randomly selected SAPALDIA participants ≥ 50 years of age. Other examinations included an interview investigating health status and measurements of blood pressure, body height, and weight. Annual exposure to NO_2_ at the address of residence was predicted by hybrid models (i.e., a combination of dispersion predictions, land-use, and meteorologic parameters). We estimated the association between NO_2_ and HRV in multivariable linear regression models. Complete data for analyses were available for 1,408 subjects.

**Results:**

For women, but not for men, each 10-μg/m^3^ increment in 1-year averaged NO_2_ level was associated with a decrement of 3% (95% CI, −4 to −1) for the standard deviation of all normal-to-normal RR intervals (SDNN), −6% (95% CI, −11 to −1) for nighttime low frequency (LF), and −5% (95% CI, −9 to 0) for nighttime LF/high-frequency (HF) ratio. We saw no significant effect for 24-hr total power (TP), HF, LF, or LF/HF or for nighttime SDNN, TP, or HF. In subjects with self-reported cardiovascular problems, SDNN decreased by 4% (95% CI, −8 to −1) per 10-μg/m^3^ increase in NO_2_.

**Conclusions:**

There is some evidence that long-term exposure to NO_2_ is associated with cardiac autonomic dysfunction in elderly women and in subjects with cardiovascular disease.

Numerous short-term studies and a few longer-term studies have linked higher air pollutant levels with increased daily morbidity and mortality from cardiovascular diseases (Forastiere et al. 2005; Hoffmann et al. 2007; Künzli et al. 2005; Le Tertre et al. 2002; Rosenlund et al. 2006). These studies mostly described the effect of particulate matter (PM) on cardiovascular health; thus, information on the effect of other pollutants (i.e., gaseous pollutants) is scarce.

The underlying biologic mechanisms linking short- or long-term exposure to air pollutants with cardiovascular disease is still a subject of research. Several hypotheses have been proposed, including direct effects of pollutants on the cardiovascular system, blood, and lung receptors, and/or indirect effects mediated through pulmonary oxidative stress and inflammatory responses (Brook et al. 2004), potentially also leading to structural changes with lasting damage of the cardiovascular system. Heart rate variability (HRV) is a measure of cardiac autonomic tone and has been described as an intermediate factor between air pollution and cardiovascular morbidity (Dockery 2001; Donaldson et al. 2001; Pope et al. 2004; Utell et al. 2002).

Associations between nitrogen dioxide and HRV have been reported but, to our knowledge, only in short-term studies (Chan et al. 2005; Liao et al. 2004; Wheeler et al. 2006). Long-term exposure to NO_2_ might lead to altered HRV through structural changes of the heart. The aim of this study was to test the hypothesis that long-term exposure to traffic-related air pollution, as measure by NO_2_ concentrations, is negatively associated with HRV in the population-based Swiss cohort Study on Air Pollution and Lung Diseases in Adults (SAPALDIA).

## Materials and Methods

This study is part of the SAPALDIA cohort study, which was originally designed to assess health effects from long-term exposure to air pollutants in the Swiss adult population. Details of its design and objectives have been reported elsewhere (Ackermann-Liebrich et al. 2005; Felber Dietrich et al. 2006). In brief, a random sample of the Swiss population was recruited from the registries of eight distinct areas. In 1991, after written invitation, a total of 9,651 participants received intensive health examinations and a detailed health interview. In 2001–2003, we were able to reexamine 8,047 of the original participants. We assessed HRV in a random selection (*n* = 1,846; 955 women, 891 men) of the 4,417 participants ≥ 50 years of age by 24-hr electrocardiograms (ECGs) after an invitation by the fieldworkers at the study center. Exclusion criteria were general or spinal anesthesia within 8 days before the ambulatory ECG recording (*n* = 5), having had a myocardial infarction within 3 months before the examination (*n* = 2), and taking digitalis (*n* = 6); no one had an artificial internal pacemaker. Further, we excluded recordings showing atrial fibrillation (*n* = 12), recordings of < 18 hr [recommendations of the Task Force of the European Society of Cardiology and the North American Society of Pacing and Electrophysiology (1996)] (*n* = 73), and recordings of insufficient quality (*n* = 6). Information on exposure to NO_2_ was missing for 267 subjects and on covariables of the model for 67 subjects. We had complete information for 1,408 subjects for analyses of the association between exposure to NO_2_ and HRV. Subjects with missing NO_2_ values did not differ from those with NO_2_ estimates (data not shown).

### HRV measurements and analyses

For the Holter recording, we used digital devices (Aria; Del Mar Medical Systems, Irvine, CA, USA) with a frequency response of 0.05–40 Hz and a resolution of 128 samples/sec. We recorded three leads (V_1_, altered V_3_ with the electrode on the left midclavicular line on the lowest rib, and altered V_5_ with the electrode on the left anterior axillary line on the lowest rib) over 24 hr. The mean ± SD duration of the Holter recordings was 22.4 ± 2.1 hr.

All recordings were scanned through a StrataScan 563 (Del Mar Medical Systems) and interpreted using the interactive method, with a final visual check on the full disclosure. We manually validated the length of each RR interval during this step. We resampled HRV at 4 Hz, and performed spectral analysis by the fast Fourier transform method. Only normal-to-normal (NN) intervals were used, with intervals excluded because of ectopy, or artifacts being replaced by holding the previous coupling interval level throughout the time interval to the next valid coupling interval. We computed 24-hr and nighttime values of the standard deviation of all normal RR (NN) intervals (SDNN), which are summary measures of HRV, as well as the following frequency domain variables: total power (TP) (≤ 0.40 Hz), low-frequency (LF) power (0.04–0.15 Hz), high-frequency (HF) power (0.15–0.40 Hz), and the LF/HF ratio. TP is an index of overall variability of heart rate, HF power is an index of the parasympathetic modulation of heart rate, and LF power is an index of the combined parasympathetic and sympathetic modulation of heart rate (North American Society of Pacing and Electrophysiology 1996; Sztajzel 2004). The LF/HF ratio shows the balance between the sympathetic and parasympathetic nervous system. Nighttime, during which HRV is less likely to be influenced by short-term disturbances, was defined as the time when subjects indicated in the diary that they where sleeping.

To avoid a biased result because of methacholine challenge, which was part of the SAPALDIA lung function testing and which, for practical reasons, was performed before the Holter recording, we excluded the first 2 hr of all recordings.

Holter recordings were made between August 2001 and March 2003. Recorders were placed on participants who had given consent after a detailed health interview. Participants were asked to follow their regular daily routine and to complete a time–activity diary during the recording period.

### Air pollutant exposure estimation

Long-term individual exposure to NO_2_ was estimated with regression models (Liu LJ, Keidel D, Gemperli A, Hazenkamp M, Bayer L, Rochat T, et al., unpublished data) for individual SAPALDIA areas that use dispersion model predictions (Liu et al. 2007) as the urban background level, parameters from the geographic information system for each residence to reflect local geographic and traffic information, time functions and meteorologic parameters to account for temporal variation, and space–time interactions. Predictors in these models varied by areas and generally included the dispersion model predictions, distance between residence and major streets, density of street network and car volumes within 200 m buffer of the home, area coverage of streets, population/apartment densities, temperature, and space–time interactions. These models predicted biweekly NO_2_ concentrations throughout 2002, which we averaged over the year to obtain annual averages. We validated the model with home out-door NO_2_ concentrations collected from a subset of the subjects; the model had an *R*^2^ between 0.77 and 0.86, depending on area (Liu LJ, Keidel D, Gemperli A, Hazenkamp M, Bayer L, Rochat T, et al., unpublished data). We cross-validated the stability of the models with results showing biases in predicted home outdoor to central site concentration ratios well below 0.02 for all areas (Liu LJ, Keidel D, Gemperli A, Hazenkamp M, Bayer L, Rochat T, et al., unpublished data). We derived spatially resolved annual concentrations of PM <10 μm in aerodynamic diameter (PM_10_) from a validated dispersion model and also assigned concentrations to residential addresses of the subjects (Downs et al. 2007). For analyzing short-term effects of NO_2_, PM_10_, and PM_2.5_, we used averaged pollution measures of the same day as the Holter recording. We used data from the nearest measurement stations of subjects’ home addresses, excluding subjects living farther than 10 km from the station or having moved in the previous year.

### Other measurements

Body height and weight were measured with participants not wearing any shoes or coats and body mass index (BMI) was calculated as weight (kilograms) divided by height (meters) squared. Exposure to environmental tobacco smoke and frequency of physical exercise was assessed by questionnaires. With the participant at rest in the sitting position, blood pressure was measured twice on the left upper arm by an automatic device (705CP; OMRON, Tokyo, Japan) according to World Health Organization recommendations (WHO 1996). We obtained blood pressure values to use in the regression model by averaging the two measurements. We defined high blood pressure as either having a systolic blood pressure ≥ 140 mmHg or a diastolic blood pressure ≥ 90 mmHg, or having answered yes to the question, “Do you have the following condition: Hypertension?” We used the highest degree of education as a proxy for social position. We measured serum levels of uric acid and total cholesterol as known cardiovascular risk factors through enzymatic tests by the Institute of Clinical Chemistry of the University Hospital of Zürich (Hitachi Modular Autoanalyser; Hitachi, Rotkreuz, Switzerland; assays from Roche Diagnostics, Mannheim, Germany).

Ethical approval for the study was given by the central Ethics Committee of the Swiss Academy of Medical Sciences and the Cantonal Ethics Committees for each of the eight examination areas, and subjects signed an informed consent at the examination. We certify that we followed all applicable institutional and governmental regulations concerning the ethical use of human volunteers and the Declaration of Helsinki during this research.

### Statistical methods

We assessed differences in proportions and means between sexes using the chi-square test and the Student’s *t*-test, respectively. Because an initial inspection suggested that the distribution of the residuals was skewed, we log-transformed HRV values for further analyses; the results are presented as percent differences between the exposure groups.

To estimate the effect of exposure to NO_2_ on HRV, we used a multivariable regression model adjusting for study site (random effects), age, education, self-reported diabetes, hypertension, smoking status, frequency of physical exercise, uric acid levels, and beta-blocker intake in the previous 30 days. Because we estimated exposure to NO_2_ for the participant’s home address, we also examined the association for subjects likely to spend more time at home, including unemployed, retired, or diseased persons. The literature has repeatedly reported higher susceptibility to air pollutants in subjects with existing cardiovascular disease (Holguin et al. 2003; Wheeler et al. 2006). We therefore also stratified our analyses according to the presence or absence of self-reported medical examination or treatment because of cardiovascular problems in the previous 12 months.

In sensitivity analyses, we included the average NO_2_ levels on the day of the Holter recording as a measure of short-term exposure to air pollution into the multivariable regression model because the associations between past exposure and HRV might be confounded by short-term effects. To focus more on the effect of traffic-related NO_2_ on HRV, we also adjusted for NO_2_ from sources other than traffic in another analysis.

We performed statistical analyses using Stata 9.2 (StataCorp, College Station, TX, USA) and SAS version 9.1 (SAS Institute Inc., Cary, NC, USA).

## Results

Table 1 shows the characteristics of the study population. Men had on average more cardiovascular risk factors than women (i.e., higher BMI, blood pressure, uric acid levels, prevalence of self-reported diabetes and of current smoking), whereas history of cardiovascular disease did not differ significantly between sexes. On the other hand, educational level of men was on average higher than that of women, and they engaged more often in exercise. One-year average exposure to NO_2_ ranged from 7 to 50 μg/m^3^, with a median of 20 μg/m^3^ and a mean of 23 μg/m^3^. Compared with SAPALDIA participants ≥ 50 years of age who did not have an HRV measurement, participants of this study less frequently had hypertension (49% vs. 63%) and self-reported diabetes (3.6% vs. 5.7%), were less frequently current smokers (19% vs. 22%), and had a higher educational level (25% with tertiary education vs. 21%).

Covariate-adjusted regression coefficients of NO_2_ exposure for different indices of HRV are given in Table 2 [crude regression coefficients are presented in Supplemental Material, Table 1 (http://www.ehponline.org/members/2008/11377/suppl.pdf)]. These were adjusted for age, BMI, hypertension, frequency of exercise, beta blocker use, uric acid, self-reported diabetes, smoking status, educational level, and random area effects. Women, but not men, showed a consistent negative association between NO_2_ exposure and HRV. Among women, each 10-μg/m^3^ increment in 1-year averaged NO_2_ level was associated with a decrement of 3% [95% confidence interval (CI), −4 to −1] in SDNN, of 6% (95% CI, −11 to −1) in nighttime LF, and of 5% (95% CI, −9 to 0) in nighttime LF/HF. Removing 2.5% of observations at each end of the NO_2_ exposure range showed similar results [Supplemental Material, Table 2 (http://www.ehponline.org/members/2008/11377/suppl.pdf)].

**Figure 1:**
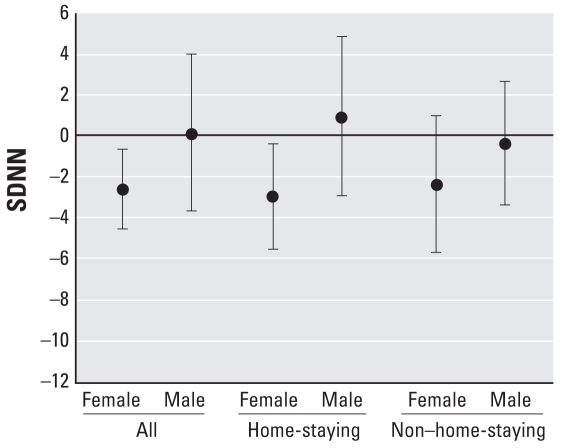
Estimated percent difference and 95% CI in SDNN per 10-μg/m^3^ increment in the annual mean NO_2_ stratified by time spent at home, adjusted for age, BMI, hypertension, exercise, beta blockers, uric acid, self-reported diabetes, smoking status, educational level, and random area effects.

To assess possible reasons for the observed difference between sexes and because women spend more time at home (Table 1), we further examined the association between exposure to NO_2_ and HRV for subjects likely to spend more time at their home address. Figure 1 shows the covariate-adjusted percent differences in SDNN per 10-μg/m^3^ increment in mean NO_2_ among home-staying and non–home-staying men and women. A significant association between exposure to NO_2_ at the home address and HRV can be seen only in home-staying women: 24-hr SDNN was 3% (95% CI, −6 to −0.4) lower per 10-μg/m^3^ increase in NO_2_, and TP was 9% (95% CI, −15 to −3) lower. Home-staying men showed no such association [for complete data, see Supplemental Material, Table 3 (http://www.ehponline.org/members/2008/11377/suppl.pdf)].

**Figure 2:**
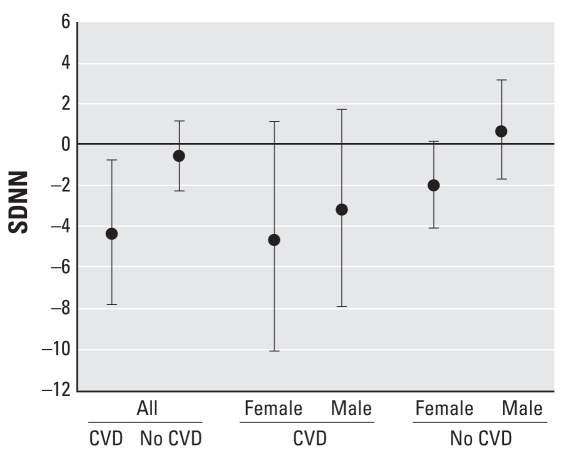
Estimated percent difference in SDNN and 95% CI per 10-μg/m^3^ increment of the annual mean NO_2_ stratified by cardiovascular disease (CVD)*^a^* adjusted for sex, age, BMI, hypertension, exercise, beta blockers, uric acid, self-reported diabetes, smoking status, educational level, and random area effects. ***a***Subjects who had a self-reported medical examination or treatment because of cardiovascular problems in the previous 12 months.

Stratification by cardiovascular disease showed that subjects who had a medical examination or treatment because of cardiovascular problems in the previous 12 months had a 4% (95% CI, −8 to −1) lower SDNN per 10-μg/m^3^ increase in NO_2_. Subjects with-out self-reported cardiovascular problems in the previous 12 months did not show a significant negative association between HRV and long-term exposure to NO_2_. When further stratifying by sex, this association was stronger in women than in men, but this difference was not statistically significant, with only 115 women and 121 men in this category (Figure 2).

Inclusion of short-term exposure to NO_2_ into the multivariable regression model (Table 3) did not change the results for long-term exposure to a relevant degree. Estimates of the short-term effect of NO_2_, PM_10_, or PM_2.5_ on HRV were all nonsignificant [see Supplemental Material, Tables 4–6 (http://www.ehponline.org/members/2008/11377/suppl.pdf)].

Additional controlling for NO_2_ from sources other than traffic at the home address did not change the results significantly (Table 4). Previous year’s PM_10_ did not show a significant association with HRV. Also, controlling for exposure to environmental tobacco smoke or gas cooking did not alter the relation between NO_2_ and HRV (data not shown).

## Discussion

This is the first study to describe effects of long-term exposure to NO_2_ on HRV in a general population sample of middle-age to elderly persons. Our results suggest that exposure to ambient air concentrations of NO_2_ averaged over 1 year is negatively associated with autonomic cardiac dysfunction in women and subjects with cardiovascular disease. Higher exposure to NO_2_ was associated with a reduction in 24-hr overall HRV and in nighttime LF power, which is influenced by both the sympathetic and parasympathetic nervous system. Because we saw no effect of NO_2_ on HF power, which is influenced by the parasympathetic nervous system (North American Society of Pacing and Electrophysiology 1996; Sztajzel 2004), the adverse effects of ambient NO_2_ on cardiovascular health might primarily involve pathways over the sympathetic nervous system.

We found a negative association between exposure to ambient NO_2_ and HRV inwomen, but not in men, that is not due to extreme observations. In the literature, findings on the modification of the effect of air pollution on cardiovascular health by sex are heterogeneous. Some earlier studies have pointed to a higher susceptibility to the effects of air pollution in females (Chen et al. 2005; Künzli et al. 2005; Rosenlund et al. 2006; Zeka et al. 2006), some did not find modification of the effect by sex (Cakmak et al. 2006; Forastiere et al. 2005; Krewski et al. 2005), and others found even a higher susceptibility in males (Hoffmann et al. 2006, 2007; Maheswaran and Elliott 2003). Having considered only residential exposure data, we examined whether our sex-specific results were confounded by behavioral differences. In subgroup analyses including only subjects likely to spend more time at their home address, we still found the association only in women, and not in men. However, studies in neighboring Germany have shown that elderly men spend less time at home than do elderly women because of gender-specific division of duties (Blanke et al. 1996; Küster 1998). Other differences between employed women and women who spend more time at home that might explain our results (e.g., level of stress) could not be addressed in this study, and we cannot rule out that these findings were due to chance.

Previous studies have suggested that subjects with underlying cardiovascular disease are at greater risk of severe events induced by air pollution (i.e., hospitalization for congestive heart failure, fatal coronary events, or adverse outcomes after myocardial infarction) (Forastiere et al. 2005; Schwartz et al. 2005; Wellenius et al. 2005; Zanobetti and Schwartz 2007). In subgroup analyses, we found an effect of long-term exposure to ambient NO_2_ only in subjects who had a medical examination or treatment because of cardiovascular problems, suggesting a higher susceptibility of subjects with an underlying cardiovascular disease. Those subjects are also more likely to spend more time at home, but in our cohort the number of women with cardiovascular problems was too small to investigate this question.

Potential mechanisms supporting our findings, including the differences in males versus females, center around the fact that traffic exposure, for which NO_2_ is a marker, or even NO_2_ by itself, might lead to chronic autonomic dysfunction through the multiple pathways that have been associated with air pollution exposure (Brook et al. 2004). Specifically, chronically elevated pulmonary and systemic inflammation may alter autonomic dysfunction. Elevated C-reactive protein has been linked to reduced HRV in the literature (Madsen et al. 2007; Park et al. 2005). Although one cannot rule out differences in physiologic responses to air pollution between sexes [differences between the sexes have been noted in response to cigarette smoking (Gan et al. 2006)], we believe that it is more likely that the main explanation for the effect differences by sex is that exposure misclassification for women who spend more hours at home is smaller than for men who travel.

Despite some limitations, our personal exposure assessment has several advantages compared with previously reported studies of long-term exposure to air pollution. Most earlier studies assigned exposure estimates to groups of individuals residing in the same city or close to the same pollution monitor, thus providing less differentiation.

In a sensitivity analysis, we included short-term exposure to NO_2_ into the model. The results did not sizably change compared with the model without short-term NO_2_, indicating that the reported results reflect a long-term effect. Unlike several panel studies, we found no association between same-day air pollution levels and HRV. However, our study design, where we measured HRV cross-sectionally, is not optimal for answering this question. For comparison, we used the same model for investigating short-term as well as long-term effects, although the two analyses would probably require different confounders to be considered (e.g., seasonal terms and meteorologic variables). We also analyzed the effect of road-traffic–related NO_2_ on HRV in a further sensitivity analysis and found similar results as in the baseline analysis. If NO_2_ were serving primarily as a surrogate for ultrafine particles, then we would expect that removing the part of the NO_2_ association that is due to regional sources would increase the effect size. This was not the case, which suggests that the effect may be due to NO_2_ itself. Confounding by indoor sources of NO_2_ is also unlikely because controlling for environmental tobacco smoke or gas cooking did not modify the relation between NO_2_ and HRV.

## Conclusions

We found some evidence that long-term exposure to NO_2_ is negatively associated with cardiac autonomic dysfunction in middle-age to elderly women and in subjects with cardiovascular disease. The different associations in men and women might be at least in part due to confounding by behavioral differences between the sexes.

## Figures and Tables

**Table 1:** Characteristics of the study population.

Characteristic	Men (*n* = 683)	Women (*n* = 725)
NO_2_, 1-year average (μg/m^3^)	22.7 ± 0.36	22.7 ± 0.35
NO_2_, same day (μg/m^3^)	25.4 ± 0.62	24.5 ± 0.61
PM_10_, same day (μg/m^3^)	23.9 ± 0.68	23.4 ± 0.68
PM_2.5_, same day (μg/m^3^)	20.0 ± 0.75	18.6 ± 0.71
Exposed to environmental tobacco smoke [no. (%)]	153 (22.4)	144 (19.86)
Cook with gas [no. (%)]	45 (6.6)	57 (7.9)
Age (years)	60.2 ± 6.0	60.4 ± 6.3
Tertiary education [no. (%)]	248 (36.3)*	105 (14.5)
Home-staying^a^ [no. (%)]	230 (33.7)**	402 (55.5)
BMI (kg/m^2^)	27.1 ± 3.5*	26.2 ± 4.9
Systolic blood pressure (mmHg)	137 ± 19*	127 ± 19
Diastolic blood pressure (mmHg)	84 ± 11*	79 ± 10
Have history of hypertension [no. (%)]	367 (46.3)*	315 (43.5)
Have self-reported diabetes [no. (%)]	32 (4.7)	22 (3.0)
Uric acid (μmol/L)	365 ± 78*	285 ± 68
Cholesterol (mmol/L)	6.2 ± 0.0**	6.4 ± 0.0
Exercise (> 1 hr/week)	337 (49.3)*	280 (38.6)
Are current smokers [no. (%)]	205 (30.0)*	133 (18.3)
Use beta blockers [no. (%)]	78 (11.4)	81 (11.2)
Have known cardiac disease^b^ [no. (%)]	121 (17.7)	115 (15.9)
Take diuretics, sympathomimetics, calcium channel blockers, angiotensin-converting enzyme inhibitors	92 (13.5)	110 (15.2)

Values shown are mean ± SD except where indicated.

a Unemployed, retired, or diseased persons.

b Self-reported medical examination/treatment because of cardiovascular problems in the 12 months before the ECG.

* *p* < 0.001, and

** *p* < 0.01 for difference between sexes.

**Table 2:** Adjusted regression coefficients^a^ of annual home outdoor exposure to NO_2_^b^ (by 10 μg/m^3^) in models of indices of HRV, by sex.

	Males (*n*= 683)				Females (*n*= 725)			
	24-hr		Night		24-hr		Night	
Outcome variable	Coefficient (SE)	*p*-Value	Coefficient (SE)	*p*-Value	Coefficient (SE)	*p*-Value	Coefficient (SE)	*p*-Value
ln(SDNN)	0.0008 (0.011)	0.946	0.0109 (0.014)	0.429	−0.0256 (0.010)	0.012	−0.0145 (0.012)	0.268
ln(TP)	0.0137 (0.027)	0.617	0.0049 (0.030)	0.870	−0.0545 (0.029)	0.074	−0.0351 (0.024)	0.186
ln(HF)	0.0131 (0.042)	0.759	0.0138 (0.045)	0.763	−0.005 (0.043)	0.910	−0.0051 (0.038)	0.896
ln(LF)	0.0046 (0.031)	0.882	−0.0112 (0.031)	0.721	−0.0347 (0.030)	0.261	−0.0663 (0.028)	0.019
ln(LF/HF)	−0.0079 (0.027)	0.768	−0.0254 (0.027)	0.353	−0.0253 (0.025)	0.317	−0.0505 (0.025)	0.043

a Adjusted for age, BMI, hypertension, exercise, beta blockers, uric acid, self-reported diabetes, smoking status, educational level, and random area effects.

b Averaged over the previous year.

**Table 3:** Regression coefficients^a^ of annual home outdoor exposure to NO_2_^b^ (by 10 μg/m^3^) in models of indices of HRV, adjusting for short-term exposure to NO_2_.

	Males (*n* = 586)				Females (*n*= 602)			
	24-hr		Night		24-hr		Night	
Outcome variable	Coefficient (SE)	*p*-Value	Coefficient (SE)	*p*-Value	Coefficient (SE)	*p*-Value	Coefficient (SE)	*p*-Value
ln(SDNN)	−0.0200 (0.015)	0.174	−0.0076 (0.017)	0.653	−0.0343 (0.014)	0.015	−0.0160 (0.015)	0.304
ln(TP)	−0.0458 (0.035)	0.189	−0.0167 (0.037)	0.652	−0.0413 (0.035)	0.243	−0.0131 (0.032)	0.683
ln(HF)	−0.0486 (0.053)	0.360	−0.0281 (0.055)	0.612	0.0286 (0.053)	0.594	0.0214 (0.048)	0.659
ln(LF)	−0.0421 (0.040)	0.309	−0.289 (0.042)	0.491	−0.0083 (0.039)	0.833	−0.0215 (0.038)	0.572
ln(LF/HF)	0.0075 (0.034)	0.825	0.0030 (0.036)	0.932	−0.0224 (0.032)	0.487	−0.0328 (0.034)	0.335

a Adjusted for same-day average NO_2_ exposure, age, BMI, hypertension, exercise, beta blockers, uric acid, self-reported diabetes, smoking status, educational level, and random area effects.

b Averaged over the previous year.

**Table 4:** Regression coefficients of annual home outdoor exposure to road-traffic–related NO_2_^a^ (by 10 μg/m^3^) in models of indices of HRV.

	Males (*n* = 683)				Females (*n*= 725)			
	24-hr		Night		24-hr		Night	
Outcome variable	Coefficient (SE)	*p*-Value	Coefficient (SE)	*p*-Value	Coefficient (SE)	*p*-Value	Coefficient (SE)	*p*-Value
ln(SDNN)	0.0053 (0.011)	0.631	0.0131 (0.013)	0.320	−0.0247 (0.010)	0.009	−0.0175 (0.012)	0.145
ln(TP)	0.0181 (0.026)	0.579	0.0140 (0.029)	0.630	−0.0577 (0.026)	0.034	−0.0279 (0.022)	0.205
ln(HF)	0.0199 (0.039)	0.622	0.0277 (0.043)	0.525	0.0005 (0.039)	0.991	−0.0084 (0.035)	0.811
ln(LF)	0.0162 (0.029)	0.581	0.0026 (0.031)	0.935	−0.0272 (0.027)	0.331	−0.0554 (0.026)	0.035
ln(LF/HF)	−0.0034 (0.026)	0.894	−0.0251 (0.026)	0.337	−0.0220 (0.023)	0.339	−0.0395 (0.023)	0.089

a Adjusted for NO_2_ from non–road-traffic sources (household, industry, agriculture/off-road, and background) averaged over previous year, age, BMI, hypertension, exercise, beta blockers, uric acid, self-reported diabetes, smoking status, educational level, and random area effects.
